# Ovarian Strumal Carcinoid: Case Report, Systematic Literature Review and Pooled Analysis

**DOI:** 10.3389/fendo.2022.871210

**Published:** 2022-04-21

**Authors:** Antonella Turla, Manuel Zamparini, Massimo Milione, Salvatore Grisanti, Vito Amoroso, Rebecca Pedersini, Deborah Cosentini, Alfredo Berruti

**Affiliations:** ^1^ Department of Medical and Surgical Specialties, Radiological Sciences, and Public Health, University of Brescia, Medical Oncology, ASST Spedali Civili, Brescia, Italy; ^2^ 1st Pathology Division, Department of Pathology and Laboratory Medicine, Fondazione Istituto di Ricovero e Cura a Carattere Scientifico (IRCCS) Istituto Nazionale dei Tumori, Milan, Italy

**Keywords:** ovarian strumal carcinoid, neuroendocrine tumors, teratomas, peptide YY, constipation

## Abstract

**Background:**

Ovarian strumal carcinoid is a rare tumor in which thyroid (struma) and carcinoid components coexist. The disease is generally considered to be a borderline malignancy, however, cases with metastatic disease have been described. No data in the literature are available to guide diagnosis and therapy.

**Methods:**

We performed a pooled analysis and a systematic review of histopathological-confirmed strumal carcinoid cases published in the literature using the following keywords: “strumal carcinoid of the ovary”, “strumal carcinoid case report”. A case of strumal carcinoid tumor diagnosed and followed-up at the Medical Oncology Unit of Spedali Civili (Brescia, Italy) was also described and included.

**Results:**

Sixty-six eligible publications were identified, providing data from one hundred and seventeen patients, plus a case diagnosed at our institution. At presentation, among the eighty-eight patients with symptomatic disease, 37% of patients suffered from abdominal distention and 49% from pain due to a growing abdominal tumor mass, 37% from constipation (peptide YY was analyzed in only nine of them, resulting above the physiologic range). Surgery was the primary therapy in 99% of the patients. Three patients had metastatic disease at diagnosis and five patients underwent recurrence after radical surgery. Histology at disease recurrence concerned the thyroid component in two patients, the carcinoid component in two patients, both histologies in one patient. Median disease-free survival and overall survival in this series were not attained.

**Conclusion:**

Strumal carcinoid of the ovary generally presents a benign behavior and surgery is curative in most cases. However, a small group of patients with this disease can undergo disease recurrence due to both the thyroid and the neuroendocrine (carcinoid) components. A follow-up in radically operated patients is therefore needed, particularly in those with a voluminous disease at diagnosis.

## Introduction

Ovarian carcinoid tumors are the most common primary neuroendocrine neoplasms in the female genital tract, and almost all arise among mature teratomas (1). Two main architectural patterns of primary ovarian carcinoids were described: insular and trabecular (2). Strumal carcinoid is a distinctive form of ovarian teratoma consisting of thyroid tissue intermixed with a neuroendocrine tumor (carcinoid) component (3), usually either insular or trabecular in type (2). It is classified as a monodermal teratoma (struma) with a secondary somatic tumor (carcinoid). The thyroid component can be normal thyroid, containing colloidal material, microfollicular or macrofollicular adenoma or papillary and follicular carcinoma. Other teratomatous elements are noted in over 80% of cases (3). The disease is extremely rare accounting for 0.1% of all ovarian malignancies (4). Strumal carcinoid is a borderline malignancy and a conservative surgical treatment, such as salpingo-oophorectomy or a simple oophorectomy, can be resolutive. However, metastases have been rarely described. The neoplastic neuroendocrine cells acquire an aggressive behavior, replacing the follicular lining cells. Rarely the thyroid component can be manifest as papillary or follicular thyroid carcinoma. Most strumal carcinoids are incidental findings; frequently they can cause symptoms due to mass enlargement and/or releasing of specific peptides and hormones. Serotonin-like substances are released directly into systemic circulation through the ovarian venous system bypassing hepatic deactivation, therefore, like all primary ovarian carcinoids, carcinoid syndrome (facial flushing, diarrhea, bronchospasm, and edema) is reported relatively frequent and has been estimated to occur in one-third of patients with insular patterns (5). Ovarian carcinoids may also typically produce and release the gut hormone peptide YY, which function is to inhibit gut motility and can cause severe constipation and painful defecation. Other hormone-related symptoms, such as hirsutism and sexual pilosity, are also described due to stimulated ovarian stroma adjacent to the tumor. Strumal carcinoid is a neoplastic pathology whose management can be difficult because of its extreme rarity and the dual thyroid and neuroendocrine component, both potentially responsible for a malignant behavior and endocrine associated syndromes. Published literature is essentially represented by case reports, small series and pathology reviews and does not provide useful information regarding the appropriate diagnostic workup and treatment and no internationally agreed guidelines are currently available.

This study was undertaken to systematically review all strumal carcinoid cases published in literature with the description of an additional patient observed in our Institution. We performed a pooled analysis to obtain information on clinicopathological features, treatment strategies and prognostic factors of this rare disease. On these bases, we provided some recommendations about the possible clinical approach.

## Patients And Methods

### Search Strategy

We performed a systematic literature search on PubMed/Embase using the following keywords: “strumal carcinoid of the ovary”, “strumal carcinoid case report”, “ovarian strumal carcinoid”; an addition search based on the main publication references was carried out. Only papers reporting individual clinicopathological data were eligible. Case reports were selected if the histological diagnosis of described cases reported the coexistence of both carcinoid tumor and strumal tissue. A restriction for language and duplicated publications was applied. A case of strumal carcinoid tumor diagnosed and followed-up at the Medical Oncology Unit of Spedali Civili (Brescia, Italy) was also included in this study.

### Statistical Analysis

Data concerning demographic, tumor sizes, histopathological features, treatments and recurrence of the disease were collected into a database; the resulted population was analyzed as a single cohort. Survival curves were obtained using the Kaplan-Meier method and compared with the log-rank test. Exploratory analyses were performed using Cox proportional hazards regression models to test the prognostic value of clinical features and treatment approaches [hazard ratios (HRs) and 95% confidence intervals (CIs)] for overall survival (OS), which is defined as the time from diagnosis to patient death or the date of the last follow-up used for censoring, and disease-free survival (DFS), which is defined as the time to relapse, second cancer, or death, whichever event came first. Patients who did not experience any of these events were censored at the reported last follow-up. P values <0.05 (two-sided) were considered statistically significant. All statistical analyses were obtained using SPSS version 23.0 (SPSS, Chicago, IL).

### Case Report

A 55-years-old white woman with no significant co-morbidities underwent an ultrasound in September 2019 for persistent constipation for several weeks associated with abdominal pain. Ultrasonography detected a cystic lesion (41 x 32 mm) on the left ovary. Transvaginal ultrasonography confirmed a dishomogeneous mass (hypoechogenic in the middle) in the left ovary while no abnormalities were found in the right ovary and the uterus. Serum CA 125 was in the normal range. A laparoscopic bilateral salpingo-oophorectomy was performed in December 2019. Pelvic and para-aortic lymph node metastases were not observed.

Histology revealed a strumal carcinoid: the first component was carcinoid tumor; the other was constituted by thyroid follicle-like tissue. At immunohistochemistry the carcinoid component was positive for chromogranin A, synaptophysin, CD56 and cytokeratin 7, while the strumal component was positive for TTF1. AFP (Alpha-Fetoprotein) expression was negative. Ki67 immunostaining was detected in about 2% of cells. The tumor was limited to the ovarian parenchyma and did not involve the capsule. Cytological examination of the ascites fluid was negative. A post-operative Gallium-68 PET/TC was negative.

Constipation resolved some weeks after the surgical intervention and at the last follow-up evaluation in October 2021 the patient was alive and disease-free.

## Results

### Search Results

Data from one hundred and eighteen patients were analyzed: one hundred and seventeen were obtained from sixty-six eligible publications, while the one hundred and eighteenth case is represented by the patient followed at our Institution. Thirteen publications were excluded because they were published as abstracts or were written in a language other than English (or the English translation was not available). Among the seventy-five records analyzed for the eligibility process, nine publications were removed for lack of individual clinical or histological data.

### Patients’ Characteristics

Patients’ characteristics are reported in **Table 1**.

**Table 1:** Patients’ characteristics and disease-related symptoms and signs.

Patient number	118
Race*	
**Caucasian**	61/104 (58%)
**Asian**	36/104 (35%)
**African-American**	7/104 (7%)
**Not available**	14/118
**Age at diagnosis (median)**	50 (range 14-78)
**Menopausal status**	
**Pre-menopause**	56/118 (47%)
**Post-menopause**	62/118 (53%)
**Median tumor size (cm)**	8 (range 1-26)
**Metastases at diagnosis**	3/118 (3%)
**Site of metastases**	
**Peritoneum**	1/118 (1%)
**Peritoneum + abdominal organs (contralateral ovary, bladder, pelvic floor) + lymph nodes**	1/118 (1%)
**Contralateral ovary + myometrium + lungs**	1/118 (1%)
Cause of diagnosis*	
**Incidental**	28/116 (24%)
**Symptomatic**	88/116 (76%)
**Not available**	2/118
Presence of symptoms and signs*	
**Visible pelvic mass at physical examination**	37/88 (42%)
**Generalized edema**	1/88 (1%)
**Heavy menstrual bleeding**	14/88 (16%)
**Amenorrhea**	6/88 (7%)
**Vaginal discharge**	4/88 (4%)
**Constipation**	33/88 (37%)
**Pain**	43/88 (49%)
**Nausea**	4/88 (4%)
**Vomiting**	4/88 (4%)
**Hirsutism**	7/88 (8%)
**Alopecia**	2/88 (2%)
**Weight loss**	6/88 (7%)
**Abdominal distention**	33/88 (37%)
**Ulcerated skin lesions**	2/88 (2%)
**Hyperthyroidism**	2/88 (2%)
**Carcinoid syndrome: flushing, diarrhoea, hypertension**	1/88 (1%)
**Hypoglycaemia**	1/88 (1%)
**Tricuspid regurgitation**	1/88 (1%)

*The common denominator of the proportions refers to the number of patients for whom the data is available.

Median age was 50 years (range 14-78). Sixty-two (53%) were post-menopausal. Among the one hundred and four patients in which this information was available, sixty-one (58%) patients were Caucasian, thirty-six (35%) Asian and seven (7%) Afro-American.

Median tumor size was 8 cm (range 1-26). Six patients (5%) presented a mass in the contralateral ovary, consisting of teratoma in four patients (6–9), clear cell adenocarcinoma in one patient (10) and endometrial cyst in the remaining one (11).

Three patients had metastatic disease at diagnosis: one patient presented with peritoneal implants (12); one patient had a disease involving lymph nodes, peritoneum and abdominal organs (contralateral ovary, bladder and pelvic floor) (13); in one case pulmonary lesions and metastases in contralateral ovary and myometrium were detected (14). In this last record the metastatic lesions in the contralateral ovary and myometrium turned out to be deposits of carcinoid (14), while in the second case the peritoneal nodules presented thyroidal compound (13). Quiñonez et al. described a peculiar case of pseudomyxoma peritonei with multiple mucinous peritoneal implants, in the absence of appendiceal lesion, associated with ovarian strumal carcinoid (12).

### Disease-Related Symptoms and Signs

As shown in **Table 1**, tumor presentation was heterogeneous. In two patients this information was not available. Twenty-eight out of the one hundred and sixteen patients in which this information was available had no symptoms (24%), while in the remaining eighty-eight patients (76%) the diagnosis of the disease was associated with the occurrence of symptoms and signs such as abdominal distention (37%), pain (49%), constipation (37%). Less often patients presented gynecological features, such as heavy menstrual bleeding (16%), amenorrhea (7%) and vaginal discharge (4%). A pelvic mass was appreciable at the physical examination in thirty-seven patients (42%). Rarely, symptoms at presentation were generalized edema (1%); nausea (4%) and vomiting (4%); hirsutism (8%); alopecia (2%); weight loss (7%); ulcerated skin lesions (2%); hyperthyroidism (2%); hypoglycaemia (1%); tricuspid regurgitation (1%). Three patients had glucagonoma syndrome, such as necrolytic migratory erythema, weight loss, anemia, and stomatitis (15–17). One patient, suffering from hypoglycemic attacks, presented concomitantly raised levels of both glucagon and insulin (18).

Peptide YY was measured in nine patients suffering from severe constipation and resulted above the reference range in all of them.

Secretin serum levels were measured in one patient, showing elevated levels (15). Circulating serotonin was analyzed in thirteen patients and resulted to be above the normal range in nine of them (5, 17–24). In one case serotonin hypersecretion caused flushing, diarrhea, hypertension (carcinoid syndrome) (5); in another case (20) serotonin hypersecretion was associated with tricuspid regurgitation, due to fibrous deposits, which required valve bioprosthesis placement. In the remaining seven cases, serotonin hypersecretion was not associated with a typical carcinoid syndrome.

Data on imaging techniques prior to surgery are available in only forty-seven patients, thirty-four were evaluated with ultrasound, twenty-six with Computed Tomography (CT) scan and ten with Magnetic Resonance Imaging (MRI). In most cases the pelvic ultrasound was the first-step imaging, followed by a CT scan and/or MRI. No patient was suspected of strumal carcinoid before surgery, including two patients who underwent biopsies to exclude neoplastic ascites (10) and lymph node (25) metastases, respectively; both were negative for neoplastic cells.

68Ga-PET was prescribed after surgery in only one patient (26); one patient performed a post-surgery 131I whole-body scan (13); in one case thyroid ultrasound and scintigraphy imaging were evaluated after the intervention (27).

### Histologic and Immunohistochemical Features

As depicted in **Table 2**, among one hundred and twelve patients in which histology was described, the strumal component was normal thyroid-like tissue in one hundred and three patients (91%), however in nine cases (9%) it was constituted by malignant tumor tissue which consisted in papillary, follicular variant of papillary or follicular carcinomas in four, three, and two patients, respectively. Among the one hundred patients in which the information was available, the carcinoid component was described as trabecular in fifty-five (55%), insular in five (5%), or a mixture of the two histotypes in forty (40%) of them. Various differentiated cells and tissues, like hair, fat, bony elements were observed in fifty patients (42%), while mucinous component, both endocervical and intestinal, was observed in twenty-four cases (20%). Concomitant adenomyosis was reported in six patients (5%). The strumal component presented focal amyloid deposits in twenty-three patients (20%) and birefringent crystals in three patients (3%), respectively.

**Table 2:** Tumor characteristics at histopathological examination.

Strumal histology*	
**Papillary carcinoma**	4/112 (4%)
**Follicular variant of papillary carcinoma**	3/112 (3%)
**Follicular carcinoma**	2/112 (2%)
**Thyroid-like tissue**	103/112 (91%)
**Not available**	6/118
Carcinoid histology*	
**Trabecular**	55/100 (55%)
**Insular**	5/100 (5%)
**Both**	40/100 (40%)
**Not available**	18/118
**Associated mucinous tumor**	24/118 (20%)
Mucinous tumor histology*	
**Endocervical**	5/9 (56%)
**Intestinal**	4/9 (44%)
**Not available**	15/24
**Associated teratoma**	50/118 (42%)
**Associated adenomyosis**	6/118 (5%)

*The common denominator of the proportions refers to the number of patients for whom the data is available.

Immunohistochemical features confirmed the dual nature of the disease. As shown in **Table 3**, the neuroendocrine markers: Neuron Specific Enolase (NSE), chromogranin A, synaptophysin and CD56 were expressed in 100% of the tumor specimens in which they were tested. As regards as thyroid-tissue markers, thyroglobulin, thyroid transcription factor 1 (TTF1), were expressed in 91% and 88% of tumors, respectively, and calcitonin, a neuroendocrine marker of the medullary thyroid component, was found to be expressed in 52% of tumors. As expected, the immunohistochemical evaluation also documented immunostaining positivity to peptide YY in 100% of the tested tumors and serotonin in 57%. Interestingly, peptides typical of the neuroendocrine differentiation of pancreatic tumors such as glucagon and pancreatic polypeptide were expressed in 80% and 100% of the strumal carcinoids tested. Moreover, CDX2, which is expressed in the nuclei of intestinal epithelial cells, was detected in three out of five strumal carcinoids in which it was assessed (60%).

**Table 3:** Immunohistochemical expression of tumor markers.

Immunohistochemical markers*	
**NSE**	13/13 (100%)
**ChromograninA**	39/39 (100%)
**Synaptophysin**	31/31 (100%)
**TTF1**	22/25 (88%)
**Thyroglobulin**	41/45 (91%)
**Calcitonin**	12/23 (52%)
**Cytokeratin20**	6/7 (86%)
**CD56**	16/16 (100%)
**Peptide YY**	9/9 (100%)
**CDX2**	3/5 (60%)
**Serotonin**	4/7 (57%)
**Glucagon**	8/10 (80%)
**Pancreatic polypeptide**	6/6 (100%)

*The common denominator of the proportions refers to the number of patients for whom the data is available.

Proliferative activity, as measured by Ki67 expression, was tested in fourteen patients and ranged between <1% to <5%.

### Treatment Strategies Adopted After Diagnosis

Surgery was the primary therapeutic approach in all patients but one (99%), even in case of metastatic disease (12–14). One patient did not undergo a surgical approach due to an early death for cardiorespiratory arrest during hospitalization due to a hypoglycaemic attack; the diagnosis of strumal carcinoid was performed at autopsy (18). Surgery consisted of salpingo-oophorectomy, which was bilateral in sixty-seven patients (57%) and unilateral in the remaining fifty (43%). Hysterectomy was performed in fifty-nine cases (50%), while lymph nodes dissection and omentectomy were performed in seven (6%) and ten patients (8%), respectively; appendectomy was reported in fourteen (12%). One patient received two courses of radiation therapy with implants before surgery (5), while another patient with pulmonary metastatic lesions was administered three cycles of chemotherapy (the regimen was not specified) before ovarian surgery (14).

Seven patients received additional therapies after surgery. Post-operative radiotherapy was performed in three cases with bilateral disease or huge masses (one case associated with invasive cervical carcinoma). The dose delivered in the pelvic region was 30 Gy in one patient (combined with chemotherapy) (28) and 70 Gy in another one (6). In one patient, due to persistent disease after surgery (multiple nodules on the liver surface, bowel and the parietal peritoneum), I3lAu was instilled into the peritoneal cavity followed by radiotherapy (29); the radiotherapy dose delivered in this third case is missing.

Three patients were addressed to adjuvant chemotherapy due to bilateral disease, voluminous lesion (median size 12cm) or concomitant invasive cervical carcinoma. Chemotherapy consisted of three or four cycles of paclitaxel associated with cisplatin (6) or carboplatin (10) or cisplatin, adriamycin and cyclophosphamide (16).

In one case chemotherapy according to the PEB scheme (cisplatin, etoposide and bleomycin) was administered after surgery due to the persistence of residual metastatic disease (13).

### Disease Recurrence and Survival Outcome

**Figure 1:**
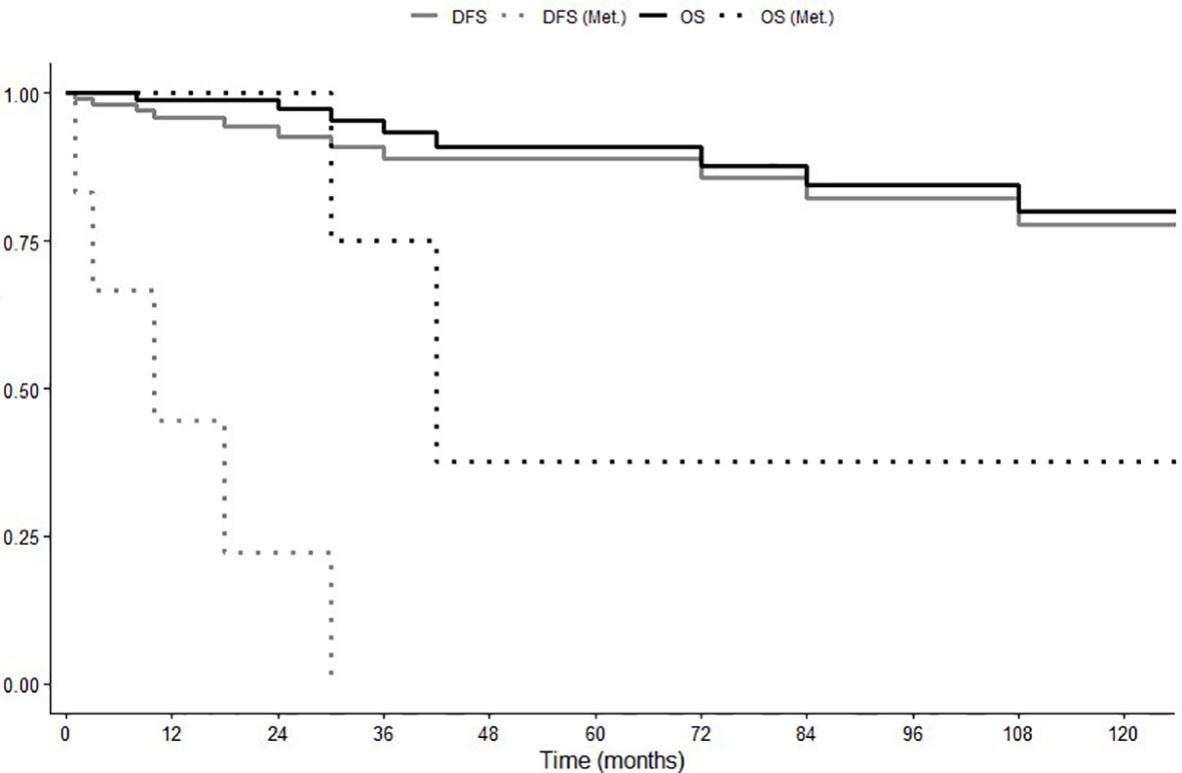
Overall Survival (OS) and Disease-Free Survival (DFS) in global population and in patients with recurrent or metastatic disease (Met.).

Follow-up data were not available in seven patients. In the one hundred and eleven patients fully assessable for prognosis, the median follow-up duration was 24 months (range 1-384 months). Three patients (3%) had metastatic disease at diagnosis, one of these was radically operated for peritoneal metastases, the remaining two did not receive radical treatment. Five patients (4.5%), radically resected, underwent disease recurrence in the follow-up. Median overall survival (OS) and median disease-free survival (DFS) were not reached (**Figure 1**). Five-year DFS was 89%, 10-year 78%; while 5-year OS was 91% and 10-year OS was 80%. Disease-related deaths were reported in two out of the nine cases (22%), both with distant metastases (11, 29), while seven patients (88%) died due to other independent causes (one patient for car accident, one for cirrhosis, one for cardiorespiratory arrest, one for rheumatic heart disease, one for cerebrovascular accident, one for intestinal necrosis, one for not specified causes).

In the five patients showing disease recurrence, this event occurred after 2 (13), 10 (17), 18 (29), 42 months (11) and 7 years (26), respectively. Four patients developed distant metastases involving peritoneum (carcinosis), breast, bone and liver; one patient underwent recurrence in the homolateral ovary 7 years after surgery and subsequently a second strumal carcinoid in the contralateral ovary almost 30 years later (26). Disease recurrence was asymptomatic (detected by follow-up imaging) in one patient (26) and symptomatic in the other four patients. The symptoms associated with the disease recurrence were: lumbago; recrudescence of constipation; amenorrhea; abdominal pain and weight loss (**Table 4**).

**Table 4:** Characteristics of patients with recurrent disease.

Recurrence of disease	5 (4.5%)
**Median age (years)**	51.5 (range 34-61)
**Median size of primary tumor (cm)**	11 (range 1.3–20)
**Recurrence-related symptoms**	
**Asymptomatic**	1/5 (20%)
**Lumbago**	1/5 (20%)
**Recrudescence of the constipation**	1/5 (20%)
**Amenorrhea**	1/5 (20%)
**Abdominal pain and weight loss**	1/5 (20%)
**Site of recurrence**	
**Locoregional (homolateral and contralateral ovary)**	1/5 (20%)
**Distant (peritoneum, breast, bone and liver)**	4/5 (80%)
**Strumal histology in primary tumor**	
**Papillary**	0
**Follicular variant of papillary**	0
**Follicular**	0
**Thyroid-like tissue**	5/5 (100%)
**Carcinoid histology in primary tumor**	
**Trabecular**	3/5 (60%)
**Insular**	0
**Both**	2/5 (40%)
**Component that drove the recurrence**	
**Carcinoid**	2/5 (40%)
**Thyroid**	2/5 (40%)
**Both**	1/5 (20%)
**Thyroid histology in metastatic thyroid-driven lesions**	
**Poorly differentiated adenocarcinoma**	1/2 (50%)
**Thyroid-like tissue**	1/2 (50%)

Due to the low number of patients who have had the event, none of the following factors, age, mucinous phenotype, tumor size, symptoms, abdominal distension, lymph node dissection + omentectomy, were associated with a significantly higher risk of disease recurrence at univariate Cox analysis. However, patients who experienced disease recurrence presented large tumor size (median size 11 cm), and, histologically, primary tumors had thyroid follicle-like structure as strumal component, while the carcinoid component was trabecular carcinoid in three patients and a mixture of trabecular and insular carcinoid in the remaining two patients. Histology at disease recurrence revealed recurrence of the thyroid component in two cases, one of them depicting poorly differentiated adenocarcinoma with areas of carcinoid (29), the second one showing well-differentiated thyroid tissue-forming follicles, similarly to the histology of primary disease (13). In two patients it was the carcinoid component that relapsed: one patient developed metastatic carcinoid in the breast (11), another patient had carcinoid recurrence in the liver associated with recrudescence of peptide YY-related constipation (17). The 5th had a peculiar disease history characterized by an initial recurrence of struma component and a second recurrence of carcinoid histology after 7 and 30 years from primary surgery, respectively (26).

As regard as treatment for metastatic disease, in the last case the double recurrence of the disease was radically removed (left adnexectomy for the first recurrence, right adnexectomy and lymphadenectomy for the second one) (26). In the remaining four cases not amenable to surgery, two of them, whose disease recurrence was thyroid histology, received thiotepa chemotherapy (29) and radioiodine therapy (13), respectively. The patient submitted to radionuclide therapy, who previously had undergone chemotherapy according to the PEB scheme for the persistence of multiple peritoneal nodules, achieved a partial remission whose duration was not specified. The patient who received thiotepa underwent disease progression at first restaging. Of the two patients, whose disease recurrence was a carcinoid tumor, one (11) had metastatic disease in breast and bone and received chemotherapy, which consisted of paclitaxel and carboplatin (three cycles) followed by etoposide and cisplatin (four cycles); both schemes administered in association with zoledronic acid. No systemic treatment was prescribed in the second case bearing liver metastases (17). This patient refused surgery and was kept in follow-up only. She was alive with progressive hepatic disease after 36 months after diagnosis suffering from drug-refractory constipation.

In the seven patients showing metastatic disease at diagnosis or experiencing disease recurrence after surgery, median OS and DFS were 42 (range 24-60) and 10 (range 0-24) months, respectively (**Figure 1**).

## Discussion

Due to its extreme rarity, little is known about the natural history of ovarian carcinoid. Therefore, no data in the literature are available to guide diagnosis and therapy.

The present series of published cases confirms that this rare disease is detected either in pre-or post-menopausal women with a slight prevalence in the latter condition. Symptoms and signs leading to the diagnosis were those related to the presence of a growing tumor mass, such as abdominal distention and pain, and those related to hormonal hypersecretion. As mentioned in the introduction, the frequent hormone-related syndromes in non-metastatic cases notoriously distinguish ovarian carcinoids from lung and gastroenteropancreatic NET (30, 31) and it is due to the hormone release directly into systemic circulation through the ovarian venous system, thus bypassing the hepatic deactivation. Among the hormone-related symptoms, constipation, as a consequence of hormone YY hyperproduction, prevailed. This is another peculiarity of ovarian carcinoids since GEP NETs do not produce YY. Noteworthy, typical carcinoid syndrome was observed in only two patients, one of them developing a carcinoid heart disease, whereas three patients developed a glucagonoma syndrome and one insulinoma syndrome; this last case was peculiar since the patient presented hyperinsulinaemic hypoglycemia and concomitant raised levels of glucagon. These data suggest that ovarian carcinoids can assume biological and clinical characteristics of both intestinal and pancreatic NETs. Amenorrhea and hirsutism, due to stimulated ovarian stroma adjacent to the tumor, were other frequent hormone-related symptoms. The thyroid component remained mostly silent in terms of hormonal-related symptoms, although two patients experienced hyperthyroidism.

Regarding the histological features, the carcinoid component had the trabecular pattern in most patients, and this is in contrast with literature data reporting insular as the most prevalent architecture in ovary carcinoids (2). These data confirm the findings of Robboy et al. which showed, in a small series of patients with strumal carcinoids, the trabecular phenotype in half of the patients (29). The strumal component in this series was mainly constituted by normal thyroid-like tissue, although malignant tumor tissue was observed in nearly 10% of cases. As expected, immunohistochemical evaluation confirms the duality of the disease, with expression of specific markers both of thyroid (thyroglobulin, TTF1, calcitonin) and carcinoid (chromogranin A, synaptophysin, NSE and CD56). The expression of peptide YY, which a typical characteristic of ovarian carcinoids, is also an expected finding. However, the positivity at immunohistochemical staining for serotonin (57% of patients), CDX-2 (60% of patients) which are markers of neuroendocrine tumors of intestinal origin, and glucagon (80%) and pancreatic polypeptide (100%) which are more typical of pancreatic NETs, was somewhat unexpected and underlines the heterogeneity of the disease and its differences from carcinoids of other districts.

The present data confirm that patients with strumal carcinoid have a favorable disease outcome, medians DFS and OS were not attained. The follow-up of this series, however, was relatively short and this is a limitation of all pooled analyses of published cases (32, 33). In most cases, the disease was diagnosed in an early stage and most patients received radical surgery. The surgical approach, however, was quite heterogeneous: all patients underwent salpingo-oophorectomy, which was bilateral in the majority of them. In about 15% of patients the operation extended to the regional lymph nodes and omentectomy, as is done in ovarian cancer. In addition, hysterectomy was performed in half of the patients. The good prognosis of this disease suggests that a conservative surgical approach can be resolutive. This issue is relevant since in many patients the disease is diagnosed in pre-menopause where the maintenance of fertility is crucial. The probable reason why extensive surgery was performed in many cases is that it was not possible to have a diagnosis of the disease pre-operatively. The assessment of hormonal production (such as peptide YY, secretin, serotonin and insulin) has to be considered when the detection of an ovarian lesion is accompanied by peculiar symptoms (constipation, flushing, diarrhea, hyper or hypoglycaemic attacks), for a pre-operative diagnosis. This is crucial since no pathognomonic imaging findings were shown. On ultrasound imaging, in fact, strumal carcinoid usually presents as a unilateral echo-mixed solid cystic mass, which often mimics a malignant tumor (34). To discriminate between benign and malignant lesions, ultrasound could be implemented with the IOTA Assessment of Different NEoplasias in the adneXa (ADNEX) RISK MODEL, which was found to be an effective diagnostic tool for preoperative evaluation of ovarian masses, resulting to be particularly useful in presence of borderline ovarian tumors (35–37). Borghese et al. applied this model showing a malignancy risk of 19.8%, above the 10% assigned as cut-off risk for malignancy (26).

It should be noted, however, that not in all cases the disease had a benign behavior. Lymph node metastases were found in two patients and the disease presented peritoneal spread in an additional one. These data underline the importance of accurate abdominal staging before surgery; the staging process should comprehend gynecologic examination with transvaginal ultrasonography, CT scan of the thorax and the abdomen and MRI in presence of suspicious liver or peritoneal metastases. More importantly, disease recurrence occurred in five strumal carcinoid patients after surgery, four of them with metastatic disease. These data suggest that a follow-up should be implemented in these patients, with periodic clinical examination and ultrasound. Second level imaging exams, such as CT scan, MRI, 68Ga PET may be prescribed as needed. Follow-up should be prosecuted for at least 5 years since most of the recurrence observed in this series occurred within 60 months. It should be noted that one patient in this series developed metastases after 7 and 30 years, suggesting a possible extension of follow-up beyond 5 years in selected cases. Due to the limited number of cases with disease recurrence, we were unable to identify baseline prognostic factors for disease recurrence.

Since there are potentially two neoplastic diseases in one, an obvious question is which of the two (thyroid carcinoma or ovarian carcinoid) drive disease recurrence. Of the five patients who relapsed, two had a carcinoid recurrence, two thyroid recurrences, while one both histologies. This observation could have a potential impact on the follow-up which should also concern the thyroid component. As pointed out in ESMO guidelines (38), isolated measurements of serum thyroglobulin cannot be reliably interpreted in the presence of normal thyroid tissue, however, the trend over time of basal thyroglobulin should be used to detect recurrent disease, and the same may be true for rising thyroglobulin Ab levels.

Noteworthy, only one of the five patients with disease recurrence regained a disease-free status after rescue surgery, all the others received systemic therapies, such as chemotherapy and 131-iodine ablation, which were scarcely effective since the only patient with recurrent thyroid component obtained a partial response after radionuclide therapy. It should be noted that patients, in which the carcinoid component drove the recurrence, received systemic chemotherapy regimens commonly used in ovarian cancer and none of them was addressed to specific therapies, such as somatostatin analogues, as recommended by international guidelines for advanced NETs (30, 31, 39, 40). Chemotherapy, in fact, is not indicated in patients with NET since they are low proliferating tumors with an indolent disease course. As a matter of fact, one of the patients in this series with metastatic disease exhibited a long survival without receiving any systemic treatment.

## Conclusion

Strumal carcinoid of the ovary represents a rare form of primary ovarian carcinoid; it can be asymptomatic, or cause symptoms related to peptide-producing components. Since most of the cases presented a benign behavior, surgical intervention is usually the only required treatment. However, a small group of patients with this disease can undergo disease recurrence due to both the thyroid and the neuroendocrine (carcinoid) components. A follow-up in radically operated patients is therefore needed, particularly in those with a voluminous disease at diagnosis. Based on the results of this pooled analysis, we propose a list of suggestions for the management of patients affected by this rare pathology (**Table 5**).

**Table 5:** Suggestions for the clinical management of patients with strumal carcinoids.

***Pre-operative diagnosis*** • Hormone work-up: YY evaluation in case of ovarian mass and severe constipation or specific hormone assessment such as insulin, glucagon, thyroid hormones, androgens, serum chromogranin A and urinary 5-hydroxy-indolil acetate according to concurrent specific syndromes. • An appropriate staging should be carried out in presence of lesions of the ovary: transvaginal ultrasound/abdominal ultrasound should be the first step, followed by CT scan of the thorax-abdomen and/or abdominal MRI. • In case of symptoms compatible with ovarian carcinoid confirmed by the hormonal assessment 68Ga PET could be useful for further diagnostic confirmation.
***Primary tumor management*** • Surgery is the mainstay of treatment. If the diagnosis of ovarian carcinoid is suspected by the specific pre-operative symptoms, a conservative intervention should be planned particularly in the case of a pre-menopausal patient. • Other treatment strategies (radiotherapy, chemotherapy) are not advisable both in neoadjuvant and adjuvant setting.
***Pathologic diagnosis*** • The criteria for the diagnosis of thyroid and neuroendocrine tumors should be adopted to define the strumal and carcinoid components.
***Follow-up, long-term implications and survivorship*** • Since few patients may experience disease relapse after surgery, follow-up with periodic clinical visits and ultrasound should be implemented. • Follow-up could include blood chemistry tests such as thyroglobulin and anti-thyroid antibodies or tests concerning the neuroendocrine component, such as YY peptide, glucagon, insulin, 5-hydroxy indolyl acetate if they were elevated in the pre-operative assessment. • CT and MRI should be prescribed in case of suspected disease progression based on clinical and laboratory findings and/or ultrasound data. • the duration of the follow-up cannot be estimated, it is reasonable that it can be at least 5 years.
***Management of advanced/recurrent disease*** • In case of local recurrence, salvage surgery should always be considered. • If disease recurrence is not amenable to surgery, it should be hormonally and/or histologically studied to determine which compound (carcinoid or thyroid) drive the disease. • As regard as the systemic therapy, specific international guidelines for NETs and thyroid tumors should be followed in case of recurrence of the carcinoid or thyroid component, respectively.

## Data Availability Statement

The raw data supporting the conclusions of this article will be made available by the authors, without undue reservation.

## Ethics Statement

Ethical review and approval was not required for the study on human participants in accordance with the local legislation and institutional requirements. The patients/participants provided their written informed consent to participate in this study. Written informed consent was obtained from the individual(s) for the publication of any potentially identifiable images or data included in this article.

## Author Contributions

AT and AB conceived the idea of this manuscript. SG, VA, RP, and DC clinically followed the patient. AT, MM, and MZ collected, interpreted the literature data and wrote the manuscript. All authors read and approved the final manuscript.

## Funding

This manuscript was supported in part by FIRM Onlus, Cremona, Italy.

## Conflict of Interest

The authors declare that the research was conducted in the absence of any commercial or financial relationships that could be construed as a potential conflict of interest.

## Publisher’s Note

All claims expressed in this article are solely those of the authors and do not necessarily represent those of their affiliated organizations, or those of the publisher, the editors and the reviewers. Any product that may be evaluated in this article, or claim that may be made by its manufacturer, is not guaranteed or endorsed by the publisher.
